# Children’s implicit recall of junk food, alcohol and gambling sponsorship in Australian sport

**DOI:** 10.1186/s12889-015-2348-3

**Published:** 2015-10-05

**Authors:** Amy Bestman, Samantha L. Thomas, Melanie Randle, Stuart D. M. Thomas

**Affiliations:** School of Health and Society, Faculty of Social Sciences, University of Wollongong, Wollongong, Australia; Australian Health Services Research Institute, Faculty of Business, University of Wollongong, Wollongong, Australia; School of Health and Social Development, Deakin University, Victoria, Australia; School of Management, Operations and Marketing, Faculty of Business, University of Wollongong, Wollongong, Australia; Legal Intersections Research Centre, University of Wollongong, Wollongong, Australia

**Keywords:** Children, Sport, Sponsorship, Advertising, Alcohol, Gambling, Junk Food

## Abstract

**Background:**

In Australia, sport is saturated by the promotion of junk food, alcohol and gambling products. This is particularly evident on player jerseys. The effect of this advertising on children, who are exposed to these messages while watching sport, has not been thoroughly investigated. The aim of this research study was to investigate: (1) the extent to which children implicitly recalled shirt sponsors with the correct sporting team; (2) whether children associated some types of sponsors with certain sporting codes more than others; and (3) whether age of the children influenced the correct recall of sponsoring brands and teams.

**Method:**

This experimental study conducted in New South Wales, Australia used projective techniques to measure the implicit recall of team sponsorship relationships of 85 children aged 5–12 years. Participants were asked to arrange two sets of magnets – one which contained sporting teams and one which contained brand logos – in the manner deemed most appropriate by them. Children were not given any prompts relating to sporting sponsorship relationships.

**Results:**

Three quarters (77 %) of the children were able to identify at least one correct shirt sponsor. Children associated alcohol and gambling brands more highly with the more popular sporting code, the National Rugby League compared to the Australian Football League sporting code. Results showed that age had an effect on number of shirt sponsors correctly recalled with 9–12 year olds being significantly more likely than 5–8 year olds to correctly identify team sponsors.

**Conclusions:**

Given children’s ability to implicitly recall shirt sponsors in a sporting context, Australian sporting codes should examine their current sponsorship relationships to reduce the number of unhealthy commodity shirt sponsors. While there is some regulation that protects children from the marketing of unhealthy commodity products, these findings suggest that children are still exposed to and recall these sponsorship relationships. Results suggest that the promotion of unhealthy commodity products during sporting matches is contributing to increased awareness amongst children of unhealthy commodity brands. Further investigation is required to examine the extent and impact of marketing initiatives during televised sporting matches on children.

## Background

Marketing and advertising practices influence the way in which children perceive products and play a role in the relationship children develop with specific products [1]. Consumer socialisation is the process where children develop consumer-related skills, knowledge and attitudes [2, 3]. This model is dependent upon the idea that age affects learning ability and that consumer socialisation is a social process that develops over a series of life stages [4, 5]. The key to this theory is that as children become more mature, their skills in decision-making and consumption intentions become more advanced from the influences of the socialising agents around them [6].

Research has shown that the way children interact with advertising and marketing can be determined based on their age [7–9]. Of note here, children younger than eight years old have been found to have very little understanding of the purpose of advertising [6, 7, 10, 11]. Instead, children this age perceive the purpose of advertisements to be informative or educational, reflecting their inability to understand advertising intent due to under-developed social cognition and information-processing skills [8, 12, 13]. They have also been found to struggle to separate television programs from the advertisements shown throughout programs [6, 14, 15]. This has led some researchers to argue that the lack of understanding with regards to advertising intent makes children of this age particularly vulnerable to marketing [16, 17].

Children between the ages of eight and twelve years have a greater understanding of the intentions of marketing, however they are still unable to identify the specific techniques used [12, 15]. At this age children have the ability to evaluate and compare products and information they receive [9, 18]. Their ability to make decisions has been found to be influenced by emotional concerns and peer influences [14, 19–21]. Children in this age group have been found to become attached to real life influences, like sports heroes or movie stars as opposed to fictional characters [9].

Marketers are increasingly using non-traditional forms of advertising to appeal to children [16, 22]. The distinction between entertainment content and advertising content is arguably more blurred in sport sponsorship, where brand imagery may be displayed continuously throughout the entertainment content [22]. Sponsorships are often viewed by marketers as a particularly persuasive advertising channel because consumers view sponsorships less sceptically than traditional marketing [23, 24]. Sponsorships can result in an image transfer for consumers where the perceptions of either the sponsor (and/or product) or the event are transferred to the other because of the sponsorship associations [25]. Most commonly in sport sponsorship, the sponsor becomes associated with a favourable attitude towards the sporting team and consumer perception of the brand is enhanced [25, 26]. The increased exposure increases the consumer’s ability to link the sponsor with the sport/team and can be easily recalled by the consumer [27].

Researchers have raised particular concerns about the sponsorship of elite sports by junk food, alcohol and gambling products (subsequently referred to here as unhealthy commodity products) due to their potential long-term negative health implications [28–30]. One implication of unhealthy commodity product advertising in sport is the normalisation of these products to children, with particular concern raised about the normalisation that may occur when children are exposed to the marketing for adult products such as alcohol and gambling [30–33]. Researchers argue that this normalisation can influence both children’s intentions to consume and their actual consumption of these products [34–42].

A few studies, mostly in the area of junk food, have examined the extent of unhealthy commodity product sponsorship of children’s sport [43], as well as of those sports with high children viewership [40, 44]. These studies show that a high proportion of sporting organisations are sponsored by unhealthy commodity products [44]. Researchers have also explored parent and community attitudes to unhealthy commodity industry sponsorship of sport, with parents in particular believing that such sponsorship positively influences children’s attitudes towards these products [45, 46].

Very few studies have examined children’s recall of and/or attitudes towards the sponsorship of sport by gambling, alcohol and junk food companies [47–49]. The most recent of these studies investigated children’s implicit recall of sports sponsors in Australia and found that although many children (aged 5–12 years) did not recall correct sponsors, they often associated a product from the same category (alcohol, junk food or control) as the correct team sponsor [49]. In Australia, we would argue that this implicit recall is compounded because of the saturation of unhealthy commodity product promotion during sport [50–53] in combination with relatively high sports’ viewing by children [54]. However, we know of no studies that have looked at children’s recall of very specific types of sponsorship such as jersey or shirt sponsors which to date do not appear to have been scrutinized as potential targets for regulation, but which are inherently tied to sports celebrities and heroes [49, 50].

Against this background, the aims of this study were to investigate:

1.  The extent to which children (aged 5–12 years) implicitly recalled shirt sponsors with the correct sporting team;
2.  Whether children associated some types of sponsors with certain sporting codes more than others; and
3.  Whether age of the children influenced the correct recall of sponsoring brands and teams.

## Methods

### Approach

This study extended upon the research of Pettigrew and colleagues [49] who used projective techniques to explore children’s implicit associations between professional sporting teams and their sponsors with a community sample of 162 children (aged 5-12 years) in Western Australia. Projective techniques involve presenting a subject with an undefined stimuli and asking them to make sense of what they see [55]. Using this method allowed children to express their conscious or unconscious attitudes towards sponsoring brands, developed through implicit or explicit messages viewed during sporting events.

### Recruitment

Children aged 5–12 years were recruited from three local junior sports competitions (Australian Football League, soccer and netball) in a regional area in New South Wales (NSW), Australia. Children and their parents were approached by researchers at sporting grounds and invited to participate in the activity. Parents were given an information sheet and gave written consent for their child to participate. Children also gave verbal consent before participating. Ethics approval was obtained from the University of Wollongong Human Research Ethics Committee.

### Data collection

Children were presented with two whiteboards. Whiteboard #1 contained seven team logos down the middle (with a unique identifier which recorded age and gender), and Whiteboard #2 contained 16 brand magnets, distributed randomly around the board. The seven sporting teams were chosen because they represented a mix of local, state, interstate and national level professional teams. These included: three National Rugby League (NRL) teams (one local team-St George Illawarra Dragons, with sponsor St George Bank (control brand), one state-based team- NSW State of Origin Blues, with sponsor Victoria Bitter (alcohol brand), and one metropolitan team in the same state- South Sydney Rabbitohs, with sponsor Crown Resorts (gambling brand)); two Australian Football League (AFL) teams (one metropolitan team in the same state-Sydney Swans, with sponsor QBE Insurance (control brand) and one based interstate-Carlton Blues, with sponsor Mars (junk food brand); one National Basketball League (NBL) team (local team-Wollongong Hawks, with sponsor McDonalds (junk food brand)); and finally the national Australian Cricket team, with sponsor Carlton Draught (alcohol brand). Of the teams included, two had shirt sponsorships with junk food brands, two were sponsored by alcohol brands and one by a casino (gambling brand). The final two teams were not sponsored by unhealthy commodity products and formed the controls for the study. Images of the whiteboards are available from the authors upon request.

In addition to the correct sponsors, a number of additional brands were placed as magnets on Whiteboard #2 as ‘dummy sponsors’. The inclusion of these brands lowered the probability of children correctly recalling sponsors through random chance and provided alternative brands for children to select from. The brands included as dummy sponsors in the study were as follows: junk food (Domino's Pizza, Oak Milk); alcohol (beer brand XXXX, wine brand Jacob's Creek); gambling related brands (Bet 365, Centrebet, the Star); control brands (Cancer Council, Commonwealth Bank of Australia). The arrangement of the team and shirt sponsor magnets on whiteboards was randomised for each experiment to reduce the likelihood of children identifying correct sponsors due to chance [49]. Consistent with the concept of projective techniques, children were not told that the activity related to sports sponsorship and were not specifically asked to ‘match’ magnets with teams [49]. Whilst children completed the activity researchers ensured that parents and friends were a reasonable distance from the child so that their results were not influenced.

Following completion of the matching activity, team and brand ‘liking’ were measured by giving children four magnets with gold stars and asking them to place them on the board next to the two brands and two teams they liked the most. Upon the completion of the magnet activity, a digital photograph of the completed whiteboard was taken. The study expanded on Pettigrew and colleagues [49], by collecting additional qualitative data from children pertaining to their rationale for placing the magnets where they did on the board, including reasons for choosing the two teams and two brands as their ‘most liked’. Furthermore, an interviewer-assisted questionnaire collected information on children’s demographics (age and gender), level of exposure to sport by asking questions relating to organised sports played, sports viewing behaviours (television and live), favourite sporting team, website viewing and ownership of branded sports merchandise.

### Approach to analysis

Gridlines were drawn onto the whiteboards prior to data collection so that researchers could objectively assess the distance between magnets when reviewing the photographs. A brand/team 'match' was identified when the brand magnet was lined up with the team magnet, within half a gridline box from the middle of the team magnet. Data was analysed using SPSS (version 19). Descriptive statistics were used to analyse the demographic characteristics of the sample including age, gender, degree of exposure to sports, the teams and brands selected as ‘most liked’ and brands most frequently associated with teams and sporting codes. Chi-square (χ^2^) tests of association were used to determine whether different groups of children (based on age and gender) differed significantly in terms of their ability to correctly match teams with sponsors.

## Results

### Sample characteristics

Of the 85 children who participated, just under half (*n* = 41, 48 %) were recruited from the junior Australian Football League competition; 34 (40 %) were recruited from the junior soccer competition; and 10 (12 %) were recruited from the junior netball competition. Children were aged from 5–12 years and had a mean age of 8.6 years (*SD* = 2.1). Just over half of the sample (*n* = 43, 51 %) was aged 5–8 years, and just under half (*n* = 42, 49 %) was aged 9–12 years. The sample was skewed towards boys (*n* = 51, 60 %). The distribution of participant ages didn’t vary between data collection venues. Participants from the junior Australian Football League and soccer competitions were predominately male. The research team selectively recruited females at the junior netball competition to ensure a comparable sample of males and females.

Almost all children (*n* = 81, 95 %) in this sample played an organised sport. About three quarters of the children (*n* = 61, 72 %) reported that they had watched sport on television in the previous week, including the National Rugby League (*n* = 44, 52 %); Australian Football League (*n* = 26, 31 %); and soccer (*n* = 21, 25 %). About two thirds (*n* = 58, 68 %) reported that they had attended a live sporting match in the previous year, including the Australian Football League (*n* = 24, 28 %); National Rugby League (*n* = 22, 26 %); and National Basketball League (*n* = 16, 19 %). Most children (*n* = 75, 88 %) reported having a favourite sporting team, including teams from the National Rugby League (*n* = 27, 32 %), soccer including a mix of international and Australian teams (*n* = 20, 24 %) and the Australian Football League (*n* = 18, 21 %). Just under half of children reported having visited a sporting team website in the previous 12 months (*n* = 38, 45 %), and the majority (*n* = 70, 82 %) reported owning branded sporting merchandise. Over a third (*n* = 31, 37 %) of children owned three or more sporting team branded items.

### Team-brand sponsorship recall

Over three quarters of children (*n* = 65, 77 %) made at least one correct recall between a sporting team and their sponsoring brand. Children aged 9–12 years were significantly more likely than children aged 5–8 years to correctly recall sponsors (*p =* 0.013). There were no significant differences in total number of sponsors recalled between boys and girls.

The most common correct recall between a specific sponsor and team was between the local National Rugby League team the St George Illawarra Dragons and its sponsor the St George bank (control) (*n* = 41, 48 %). The second most common match was between the local National Basketball League team the Wollongong Hawks and its’ sponsor McDonalds (junk food) (*n* = 28, 33 %). When examining the product relationships with teams, certain product categories were consistently placed next to specific teams. For example, while the correct sponsor relationship for the metropolitan National Rugby League team (South Sydney Rabbitohs) was Crown Resorts (gambling); one in five children (*n* = 17, 20 %) placed the Bet365 gambling product next to this team. Only three children (4 %) recalled the correct sponsor. For the state based National Rugby League team (NSW State of Origin Blues), a similar number of children (*n* = 15, 18 %) placed the Domino's Pizza (junk food) magnet next to the team, as those that recalled the correct sponsor Victoria Bitter (alcohol) (*n* = 18, 21 %). Incorrect sponsor XXXX Gold (alcohol) was another product children placed with the NSW State of Origin team, where 10 % of children associated this alcohol product with the team. In the case of Cricket Australia, 17 % (*n* = 14) of children placed Commonwealth Bank of Australia (control), an associated but not current shirt sponsor, with the team. This compared to 12 % (*n* = 10) of children who correctly recalled the current shirt sponsor, Carlton Draught (alcohol).

### Associations between sporting teams and brand categories

We also investigated whether children were able to make similar associations between teams and brands at the brand category level. Table 1 lists the sporting teams and actual (correct) sponsors in the far left hand column. The second column indicates the percentage of children who correctly matched the team with the actual sponsor. The third column shows the percentage of children who nominated that team as their ‘most liked’. The remaining four columns indicate the percentage of children who matched at least one brand from each brand category (alcohol, gambling, junk food and the control brands) with each team, with correct brand category matches indicated in bold.

**Table 1:** Number of correct brand matches, teams most liked and brand category associations by team

Team and actual sponsor (geographic region)	Correct brand match n(%)*	Team most liked n(%)*	Alcohol category matches n(%)*	Gambling category matches n(%)*	Junk food category matches n(%)*	Control category matches n(%)*
AFL team 1 Carlton Blues/ Mars (Interstate)	21 (25)	11 (13)	22 (26)	28 (33)	**37 (44)**	21 (25)
AFL team 2 Sydney Swans/ QBE Insurance (Metropolitan)	22 (26)	29 (34)	28 (33)	21 (25)	25 (29)	**44 (52)**
AFL Sporting Code Total	30 (35)	35 (41)	35 (41)	40 (47)	53 (62)	56 (66)
NRL team 1 St George Illawarra Dragons/ St George Bank (Local)	41 (48)	27 (32)	22 (26)	22 (26)	28 (33)	**43 (51)**
NRL team 2 NSW State of Origin Blues/ Victoria Bitter (State-based)	19 (22)	35 (41)	**41 (48)**	20 (24)	31 (37)	25 (29)
NRL team 3 South Sydney Rabbitohs/ Crown Resorts (Metropolitan)	3 (4)	27 (16)	33 (39)	**28 (33)**	34 (40)	16 (19)
NRL Sporting Code Total	53 (62)	63 (74.1)	69 (81)	50 (59)	62 (73)	63 (74)
Cricket Australia/ Carlton Draught (National)	10 (12)	13 (15)	**35 (41)**	23 (27)	34 (40)	23 (27)
NBL Wollongong Hawks/ McDonalds (Local)	28 (33)	28 (17)	26 (31)	16 (19)	**44 (52)**	22 (26)

^*^Percentages have been rounded to nearest whole number. Numbers in bold indicate the correct brand category match (i.e. the brand category of the actual sponsoring brand) for each team. Column and row percentages do not total to 100 % because they represent the number of children who placed the magnet category with each team. Children could place more than one product magnet with each team

As indicated in Table 1, the state-based National Rugby League team (NSW State of Origin Blues) was the team with the most number of alcohol brand matches (*n* = 41, 48 %), and was also a team sponsored by an alcohol brand. Similarly, the National Basketball League team (the Wollongong Hawks), received the highest number of junk food matches (*n* = 44, 52 %), and was also sponsored by a junk food brand. The National Rugby League team, the South Sydney Rabbitohs was associated with a gambling brand by one third (*n* = 28, 33 %) of participants, this team’s jersey sponsor was a gambling brand. The interstate Australian Football League team (Carlton Blues) was the team equally most frequently matched with gambling brands (*n* = 28, 33 %), but was actually sponsored by a junk food brand.

When comparing the brand categories most frequently matched with the two major football codes (Australian Football League and National Rugby League), more children associated both alcohol and gambling brands with the National Rugby League (whereby 81 % matched at least one alcohol brand and 59 % matched at least one gambling brand).

The Australian Football League and the National Rugby League sporting codes rows in the table indicate the percentage of children that correctly recalled, selected as ‘most liked’ or who matched one brand from the brand category (in columns four to seven) for at least one of the teams from that sporting code.

### Team and brand liking

The state-based National Rugby League team (NSW State of Origin Blues) was the team most frequently selected as ‘most liked’ by children (*n* = 35, 41 %). Of note, during the data collection period the first televised football game in which this team competed was held. Following the televised game there was a significant increase in the number of children who chose this team as one of their most liked teams (*p =* 0.004). Only three children (4 %) chose this team as their most liked prior to the game, while 32 (38 %) children selected this team as their most liked after the game was televised.

Of the top five most liked brands by children, four were junk food brands. However, one in five of the participants (*n* = 16, 19 %) selected either an alcohol or gambling brand as one of their two ‘most liked’ brands. Ten children (12 %) selected a gambling brand as one of their ‘most liked’ brands in the study. The most common reasons given by children for choosing gambling brands as their most liked were related to the image presented in the logo. For example one girl aged five stated that she chose Crown Resorts (gambling) as one of her most liked brands because “I like crowns”, whilst simultaneously pointing to the crown in the logo. Alcohol brands were selected as being most liked by seven of the participants (8 %).

### Methodological insights

A range of methodological insights emerged from this study relating to why children placed the magnets where they did on the Whiteboards. These insights are important for researchers seeking to replicate or improve upon the method developed here, and potentially add to our understanding of children’s interpretation of the research task and the way they engage with the projective techniques used. Just under one third of children (*n* = 25, 29 %) indicated that they placed the magnets next to teams that they believed the brands had a relationship with. Children used terms such as “matched them”, “put the right ones together”, and “seen them together before” to explain their rationale for arranging the magnets. For example, an eleven year old girl indicated that she had put the brands with the “teams they went for”. Thirteen children (15 %) stated that they placed magnets next to teams because of a “sponsorship” relationship. For example, an eight year old boy said he "put them there because that’s the sponsors", while a nine year old boy said “the Wollongong Hawks are sponsored by McDonald’s, I’ve watched them and memorised it”. Furthermore, a twelve year old boy who correctly matched the sponsors of six out of the seven teams (the exception being the state-based National Rugby League team) indicated that his method for arranging the magnets was to “…pick the sponsors. I put Bet365 with NSW Blues because they were on the ads more (during the game)”.

Whilst completing the magnet task, the researchers observed that some children took care and were meticulous in the way they arranged the magnets on the Whiteboard. Children who did this were from a range of age groups. They often removed magnets they had initially placed with one team and placed them elsewhere, constantly reconsidering and revising where they had placed the magnets. These were also predominantly the children who indicated that they were placing brands “with the right team” or who identified a sponsorship arrangement between the teams and the brands. Children who employed this method also placed the brand magnets in an orderly fashion, often linearly with the team magnets, and did not overlap the magnets. These children generally placed one or two brand magnets with each team or did not put any brand magnets next to a team if they could not identify a relationship between the two. Children who could not explain their rationale for magnet placement generally took less care whilst completing the task. These children frequently had overlapping magnets and brand magnets were not always straight or linear in relation to the team magnets.

## Discussion

Three key findings warrant more discussion: (1) the fact that just over three quarters of children were able to correctly match at least one sporting team with their sponsoring brand and that older children (aged 8-12 years) were more likely to correctly identify sponsors; (2) the fact that team-sponsor associations seem to be occurring at the product category level as well as the brand level, and (3) by far the most liked teams are associated with an unhealthy product (junk food), and other adult-only products are also being cited as children’s ‘most liked’. These points are discussed in more detail below.

### Team-brand sponsorship recall

Age was found to have most influence on participant’s ability to correctly match teams with sponsors. Younger children (5–8 years) were less likely to report that they placed teams and brands together because of a sponsorship arrangement than older children (8–12 years). Younger children were also less likely to complete the matching task logically ‘matching’ teams and brands together. Previous literature suggests that children (particularly those younger than eight years) lack the ability to understand embedded marketing as an advertisement [8, 10, 12, 13, 17, 22]. This would suggest that older children may be able to match more teams with sponsoring brands because they have a higher level understanding that they are seeing advertisements on player shirts, although further research is required to understand the validity of this hypothesis more fully.

### Associations between sporting teams and brand categories

At the time of data collection, the state based National Rugby League competition was highly televised and promoted. Researchers noted that the major sponsor, ‘Victoria Bitter’ (alcohol) was noticeably featured in promotional content for the competition, and research shows that this competition in particular is saturated by marketing for alcohol brands [52]. It is proposed that the high visibility of the competition and its sponsors contributed to the relatively high rate of association between alcohol brands and this team. Almost half of the children in the study implicitly associated at least one alcohol brand with the team. The alcohol brand most frequently associated with the team was the shirt sponsor ‘Victoria Bitter’. However ‘XXXX Gold’ was also highly associated with the team. The implicit association of ‘XXXX Gold’ with the state team used in this study is likely due to this brand being the long-term shirt sponsor of the opposing team in the competition the Queensland Maroons [56]. This suggests that there may be a crossover effect between the two teams where children, especially those younger than eight years old, were unable to differentiate between the major shirt sponsors of the two opposing teams.

A similar crossover effect was evident in the case of Australian Cricket team in the study whereby children more frequently associated its previous sponsor, the ‘Commonwealth Bank of Australia’, than they did the current sponsor at the time of data collection, ‘Carlton Draught’. This finding raises questions regarding the long-term effects of shirt sponsors on children and the brands associated with different teams. This finding may indicate that even if sponsorship relationships change, it may take some time for the association of the incumbent brand with the team to diminish. Limited research has been conducted into the crossover effect described above and this is an important area for further study. Literature suggests that the over commercialisation of sporting events may lead to confusion in consumers as to correct sponsors of events [57, 58]. However, this effect usually occurs in the context of ambush marketing, rather than scenarios where there are only two primary sponsors or when considering the effect of previous primary sponsors.

Future research should also investigate the temporal impacts of sponsorships on recall and likeability – for example, the degree to which children recall the correct sponsors over time – including outside of the sporting codes season. The findings of this study show that particularly older children implicitly recall at least some of the sponsors that they see aligned to sports teams, and often understand that there is a sponsorship or ‘matched’ relationship between the team and the brand. What is less clear is how this may in turn influence the consumption or desirability to consume these brands.

### Team and brand liking

Children in the study preferred local teams when asked to select the teams they liked the most. This is perhaps not unexpected, as children are more likely to be exposed to the marketing efforts of these teams at the local level (e.g. either live games or other media promotions). This has public health implications because it suggests that lower socio-demographic and disadvantaged areas, in which health problems are already more prevalent, may be doubly impacted if their local teams are all sponsored by the types of unhealthy brands which have contributed to these health issues (e.g. junk food). Future research should look to investigate whether the removal of unhealthy commodity sponsors would reduce the association of such brands with local sporting teams amongst children who live in these areas.

Targeted marketing of junk food to children has been recognised as a contributing factor to rising levels of childhood obesity in Australia, and as an important factor in the ongoing consumer socialisation of junk food as a normal part of everyday life [12, 59]. Given the age range of the sample and the extent to which junk food is marketed to children it is not surprising that children ‘liked’ junk food brands, and this result also supports previous findings that children respond positively to junk food advertising [60, 61]. However, one in five children (and children as young as 5 years old) selected an alcohol or gambling brand as their most liked. There are a number of possible explanations for this. First, the qualitative data indicated that children may just like the logos of the brands. Preference for teams and brands based on their logo was not confined to alcohol and gambling brands. Children indicated a preference for magnet logos based on their appearance for both control brands and teams as well as unhealthy commodity brands. As evident from the literature from alcohol and tobacco, children may have expressed a preference for specific brands because elements of the advertisements or logo of the brand, which often included animals and cartoons, which were found to be appealing [62, 63]. This does however have public health policy implications surrounding the appropriateness of the logos used by unhealthy commodity companies and their appeal to children. Further investigation of the appeal of unhealthy commodity sponsor logos with children is required to more fully understand this effect. If there are high appeal factors then this may support an argument for ‘plain packaging’ of  unhealthy product logos during sports matches. Second, it may be that the exposure (and normalisation) to the marketing for these brands during sport increases their 'likability' amongst children. This has been demonstrated previously with regard to the sponsorship of sport by tobacco companies where children positively perceived the sponsoring tobacco brands and were more likely to initiate brand use [64–66].

### Geographic variations

This study observed influences that were not reported on by the Pettigrew and colleages [49]. The first relates to the influence of geographic region on ability to correctly match teams and sponsoring brands. Findings indicate that children were more likely to achieve correct matches for local sporting teams compared to interstate and national teams. The three teams with the highest number of correct matches were from three separate sporting codes; however they were all from the local area and those most commonly supported by residents in the area where fieldwork was conducted.

An interesting observation in the study was that although recall was lower for the interstate team, children were still to some extent, able to recall sponsorship relationships from teams that are geographically far from their local area. This suggests that even if not through local media, young children may still be exposed to sporting matches played elsewhere via television coverage. Therefore sponsorship relationships of interstate and national teams still have an effect on the children viewing them. This underlines the importance of national regulations for sporting sponsorships as opposed to state-based regulation, because sporting coverage is often televised beyond state borders.

### Limitations

There are several limitations that should be considered when interpreting these results. One of these was that only one gambling related sponsor was used. This was because only one team in the study had a gambling sponsor (one of the National Rugby League teams). Further research should investigate into the influence of gambling sponsorships, particularly sports betting, on children. This research is necessary because of the rise in gambling brand promotions in Australian sport. Another limitation of the study was that it did not include children involved with the National Rugby League. This was because the study took a convenience sample using the most accessible teams during the period of data collection. The inclusion of children from this sporting code would have been valuable to the research because the study design included three National Rugby League teams and their sponsors. It may be the case that children who play National Rugby League have higher exposure to National Rugby League games, and thus have higher implicit recall of these sports sponsors than children not involved in National Rugby League. Consequently, not including these children in the sample may have actually resulted in an underestimation of the association between unhealthy consumption brands and the National Rugby League. This study did not consider a number of additional factors that may be interesting in terms of socio-demographic factors and brand recall. For example, it was beyond the scope of this study to examine whether correct brand match for a given team was higher for children if this was their most liked team. Finally, the study was limited by exploring sponsorship relationships with just seven teams (four from the National Rugby League sporting code) across four sporting codes. Further study is required to determine the extent to which children from different geographical areas and different sporting exposure levels form relationships with sponsors.

Despite these limitations, there were a number of key strengths of the study. One was that data was collected in a regional area from children across three different sporting codes (Australian Football League, netball and soccer) with varying levels of exposure to sport (and its’ sponsors). The second strength of this study was that by utilising a method that children engaged with, researchers were able to explore in depth the implicit relationships that children developed with sponsors and sporting teams.

## Conclusion

The increase in unhealthy commodity brands sponsoring sport is an area that has received much public attention. To date, there has been limited research analysing sport sponsorship across multiple sporting codes or the sponsorship of unhealthy commodity brands on player shirts. This study has shown the extent to which children aged 5–12 years are implicitly aware of professional sporting team sponsorships. This provides evidence that sport sponsorships may be contributing to a consumer socialisation process whereby children, through repeated and sustained exposure to unhealthy commodity brands during professional sporting matches, begin to see them more favourably and whereby these brands are normalised as part of everyday life.
